# A preschool-based intervention for Early Childhood Education and Care (ECEC) teachers in promoting healthy eating and physical activity in toddlers: study protocol of the cluster randomized controlled trial PreSchool@HealthyWeight

**DOI:** 10.1186/s12889-019-6611-x

**Published:** 2019-03-07

**Authors:** Nicole Toussaint, Martinette T. Streppel, Sandra Mul, Anita Schreurs, Marielle Balledux, Karen van Drongelen, Mirka Janssen, Ruben G. Fukkink, Peter J. M. Weijs

**Affiliations:** 1 Faculty of Sports and Nutrition, Amsterdam University of Applied Sciences, Dokter Meurerlaan 8, Amsterdam, SM 1067 The Netherlands; 2 Childcare organization Impuls, Sam van Houtenstraat 74, Amsterdam, JP 1067 The Netherlands; 3 Netherlands Youth Institute, Catharijnesingel 47, Utrecht, GC 3511 The Netherlands; 4 The Netherlands Nutrition Centre, Bezuidenhoutseweg 105, The Hague, AC 2594 The Netherlands; 5 Faculty of Child Development and Education, Amsterdam University of Applied Sciences, Wibautstraat 2-4, Amsterdam, GM 1091 The Netherlands; 6 Faculty of Social and Behavioural Sciences, University of Amsterdam, Nieuwe Achtergracht 127, Amsterdam, WS 1018 The Netherlands; 7 Department of Nutrition & Dietetics, Internal Medicine, Amsterdam University Medical Centers, location VUmc, De Boelelaan 1117, Amsterdam, HV 1081 The Netherlands

**Keywords:** Lifestyle intervention, Overweight, Obesity, Prevention, ECEC teachers, Preschool, Toddlers, Dietary intake, Physical activity

## Abstract

**Background:**

Interventions to prevent overweight and obesity in toddlers are needed to minimize health inequalities, especially in migration and lower socio-economic groups. Preschools are identified as important environments for interventions to prevent overweight and obesity. Early Childhood Education and Care (ECEC) teachers in preschools are potential key actors in promoting healthy eating and physical activity. This paper describes the research design of a Dutch preschool-based intervention for ECEC teachers in promoting healthy eating and physical activity in toddlers.

**Methods:**

PreSchool@HealthyWeight concerns a cluster randomized controlled trial on preschools in Amsterdam Nieuw-West, Netherlands. This city district is characterised by inhabitants with a migration background and low socio-economic status. Forty-one preschools, with 115 ECEC teachers and 249 toddlers/parents, were randomly allocated to an intervention or control group. An intervention for teachers will be carried out on intervention locations and consists of modified versions of 2 existing programs: ‘A Healthy Start’ and ‘PLAYgrounds’. In ‘A Healthy Start’, ECEC teachers learn to provide a healthy and active environment for toddlers. The ‘PLAYgrounds for Toddlers’ program, coaches ECEC teachers to stimulate physical activity in the playgrounds of preschools. PreSchool@HealthyWeight aims to evaluate the effectiveness of the intervention after 9 months. Primary outcomes are the teachers’ knowledge, attitude and practices concerning healthy eating and physical activity, and consequently the level of confidence of ECEC teachers in promoting healthy eating and physical activity in toddlers. Secondary outcomes include the Body Mass Index, body composition, dietary intake and physical activity level of teachers and toddlers. In addition, the activating role of ECEC teachers and the physical activity of toddlers on the playgrounds will be evaluated. Lastly, the knowledge, attitude and practices of parents concerning healthy eating and physical activity will be assessed.

**Discussion:**

It is hypothesized that this preschool-based intervention for ECEC teachers improves the knowledge, attitude and practices regarding healthy eating and physical activity, and consequently the level of confidence of ECEC teachers in promoting healthy eating and physical activity of toddlers. The intervention addresses the call for early intervention to prevent overweight and obesity and to minimize health inequalities.

**Trial registration:**

Netherlands Trial Register (NTR): NL5850. Date registered: August 26, 2016.

## Background

Although in high-income countries the increase in the prevalence of overweight and obesity among children seems to level off, the prevalence of excessive weight in children remains high and is higher in urban settings [1]. In the Netherlands, 18% of the children in the 4 largest cities were overweight in 2015 compared to 11% in other parts of the country. This difference is related to the relatively high number of families with a migration background and low socio-economic status in urban surroundings [2]. Even very young children (3 to 4.5 years old) show consequences of health inequalities, and these inequalities widen with increasing age [3–5]. The period between age 2 and 6 is important for the development of adult overweight [6]. Overweight or obese children are more likely to become overweight or obese as adults [7]. Moreover, they have a higher risk of developing non-communicable diseases and psychosocial impairments earlier in life [8]. Unhealthy eating and low physical activity levels are important causes of excessive weight gain [9]. Therefore, interventions to promote healthy eating and physical activity in toddlers are needed, especially in migration and lower socio-economic groups to minimize health inequalities.

In the Netherlands, urban preschools provide an opportunity to reach many toddlers and their parents, particularly those with a migration background or low socio-economic status. When a toddler is at risk for language or developmental delays, parents are advised to enrol their toddler in a preschool that provides play-based education. These preschools prepare children for primary school [10]. Toddlers spend up to 15 h per week in preschool and preschools are therefore identified as important environments for interventions to prevent overweight and obesity in young children [11].

Early Childhood Education and Care (ECEC) teachers in preschools are potential key actors for promoting healthy eating and physical activity in toddlers. ECEC teachers may set a healthy example and be positive role models in healthy eating and physical activity behaviours [12, 13]. In the light of this potential role, the knowledge, attitude and practices regarding healthy eating and physical activity, and consequently the level of confidence of ECEC teachers in promoting healthy eating and physical activity in toddlers is of interest.

Literature on the role of ECEC teachers in promoting healthy eating and physical activity among young children is inconclusive. Ward et al. describe in a systematic review that the effect of specific practices of childcare educators on healthy eating and physical activity in young children is not clear because of the lack of high-quality intervention trials. The authors give recommendations for future research, among which to study previously assessed practices of educators on more diverse populations [14]. Furthermore, Wolfenden et al. emphasizes in a Cochrane review that further research is needed for successful strategies to improve childcare service staff knowledge and attitudes regarding healthy eating and physical activity and to find out more about the effect of such strategies on child physical activity and weight status [15]. Our study, PreSchool@HealthyWeight (PS@HW), involves preschools in Amsterdam Nieuw-West, the Netherlands. Amsterdam Nieuw-West is characterised by inhabitants with a migration background and low socio-economic status. PS@HW intends to contribute to evidence-based best practices for ECEC teachers in diverse populations.

The primary objective of PS@HW is to increase the knowledge, attitude and practices of ECEC teachers regarding healthy eating and physical activity, and consequently the level of confidence in promoting healthy eating and physical activity of toddlers. The secondary objective is to gain insight in the effect of the preschool-based intervention on:

- Body Mass Index (BMI) and body composition of ECEC teachers and toddlers;
- dietary intake and physical activity levels of ECEC teachers and toddlers;
- the activating role of ECEC teachers regarding physical activity of toddlers on the playground;
- amount and type of physical activity in toddlers on the playground;
- motor development of toddlers;
- knowledge, attitude and practices of parents regarding healthy eating and physical activity of toddlers;
- weight perception of parents concerning the weight status of their toddler.

## Methods

### Study design and setting

**Fig. 1:**
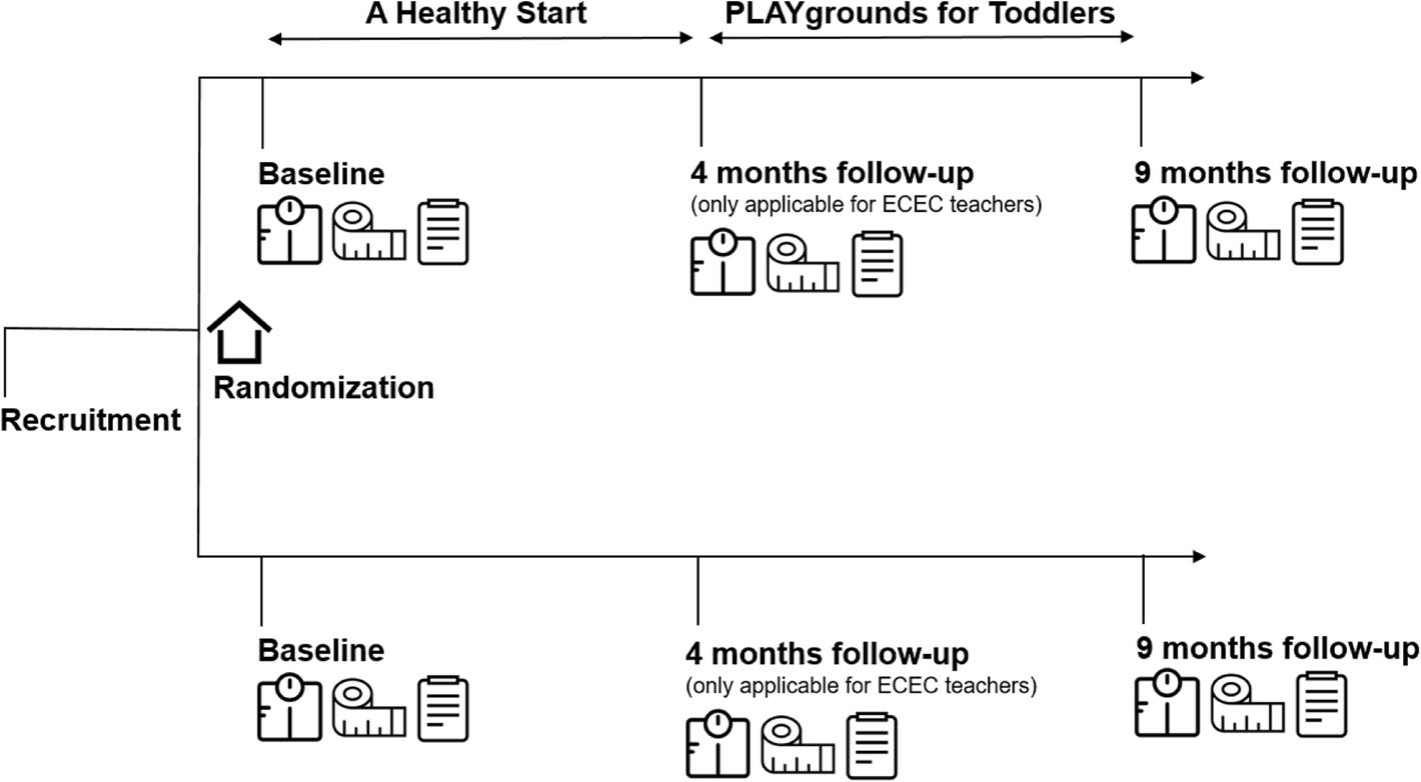
Schematic overview of the study

PS@HW concerns a cluster randomized controlled trial. Preschools of child care organization Impuls in Amsterdam Nieuw-West, the Netherlands, were randomly allocated to an intervention or control group by an independent researcher (with the use of computer-generated randomization lists). The study period is 9 months, starting with baseline measurements and ending with close-out measurements. For ECEC teachers, additional data will be collected after 4 months of follow-up. After the baseline measurements, a preschool-based intervention regarding healthy eating and physical activity will be carried out on preschools in the intervention group. The intervention consists of modified versions of 2 existing Dutch programs for ECEC teachers: ‘A Healthy Start’ (Dutch: Een Gezonde Start) and ‘PLAYgrounds’. Figure 1 provides a schematic overview of the study.

### Recruitment

In total, 42 preschools of child care organization Impuls were available for allocation. One location was excluded because of practical reasons and 4 preschools were combined in the allocation as they shared a building or playground. The final sample includes 41 preschools, in which 115 ECEC teachers and 249 toddlers and their parents were recruited. ECEC teachers who worked at multiple preschool locations were excluded. Toddlers and their parents were included if the toddler was 2.5 to 3.5 years old. ECEC teachers and parents received oral and written information about the study. For ECEC teachers, information meetings were organized. Parents were individually approached by the research staff. All participating ECEC teachers and parents gave written informed consent. Parents gave additional written informed consent for their toddlers.

### Intervention

The preschool-based intervention for ECEC teachers consists of modified versions of the programs ‘A Healthy Start’ and ‘PLAYgrounds’.

#### A Healthy Start

The national program ‘A Healthy Start’ is an initiative of the Dutch Ministry of Health, Welfare and Sport and the Ministry of Social Affairs and Employment. It is developed by 7 renowned Dutch knowledge centres (Knowledge Centre for Sports Netherlands, Netherlands Youth Institute, Pharos Dutch Centre of Expertise on Health Disparities, the Netherlands Nutrition Centre, National Institute for Public Health and the Environment, the Netherlands Organisation for applied scientific research TNO, and VeiligheidNL). Train-the-trainer courses are organized for ECEC teachers and ECEC teachers in training. ‘A Healthy Start’ focusses on the development of knowledge and practices about nutrition, physical activity and education of ECEC teachers. The national program consists of a basic model and 4 in-depth modules. The basic module includes 3 face-to-face meetings of 2.5 h each. The in-depth modules, each between 4 and 6 h, are optional and concern the themes Nutrition, Physical Activity, Weight and Safe Behaviour & Safe Environment. Special attention is given to the diversity of children and their parents [16]. For the intervention of PS@HW, research staff and 3 coaches of child care organization Impuls, who met criteria of the train-the-trainer course for the national program, composed a modified version of ‘A Healthy Start’. The modified version includes theory and practical assignments of the basic module and in-depth modules Nutrition, Physical Activity and Weight. Three face-to-face meetings of 2 h will be organized for 8 groups of ECEC teachers (in total 24 meetings). Each meeting will be led by a coach of child care organization Impuls and a member of the research staff. In the first meeting ECEC teachers will reflect on their own lifestyle and on the topic healthy lifestyle in their organization. Furthermore, theory about the Dutch food-based dietary guidelines (2015) will be discussed. The second meeting focusses on the interaction with toddlers and a healthy lifestyle, and the role of ECEC teachers in setting a healthy example. In the third meeting, the interaction with parents and a healthy lifestyle will be discussed.

#### PLAYgrounds for Toddlers

The initial ‘PLAYgrounds’ program concerns a physical education based playground program for primary schools to improve physical activity levels of children during recess times. The program consists of a multi-component alteration of the schools’ playground. In addition, the playground usage is stimulated by altered time management of recess times, teachers stimulating physical activity and a modification of the physical education content. ‘PLAYgrounds’ is proven to be effective in primary schools [17, 18]. For PS@HW, the program was adapted to the context and target group on preschools of child care organization Impuls. This ‘PLAYgrounds’ program for toddlers focusses on the amount and type of physical activity and the prompts of ECEC teachers within the possibilities of the outdoor space and availability of material in preschools. Two training sessions and 1 evaluation session will be organized for in total 4 groups of ECEC teachers. In addition, a trainer will visit each preschool in the intervention group between the training sessions to give specific instructions for improvement (coaching on the job) to the local team of ECEC teachers.

In PS@HW, ‘A Healthy Start’ and ‘PLAYgrounds for Toddlers’ have a complementary role. The multi-component and multilevel programs share a focus on providing a healthy and active environment for toddlers and the role of ECEC teachers to set a healthy example. The interventions will be applied consecutively, in order not to overload ECEC teachers. Attendance records will be used to determine the adherence of ECEC teachers to the intervention. All participants who will attend at least 2 meeting of ‘A Healthy Start’ and 1 meeting of ‘PLAYgrounds for Toddlers’ will receive certificates.

### Procedures and outcome measures

ECEC teachers and parents will be asked to fill in a questionnaire at baseline, 4 months follow-up (only applicable for ECEC teachers) and 9 months follow-up (close-out). At the same time, anthropometric measurements in ECEC teachers and toddlers will be carried out. Furthermore, observations on the playgrounds will be performed at 4 and 9 months follow-up. The questionnaire for ECEC teachers includes questions on knowledge, attitude and practices regarding healthy eating and physical activity, and questions on the level of confidence in promoting healthy eating and physical activity in toddlers. For parents, the questionnaire includes questions on knowledge, attitude and practices regarding healthy eating and physical activity of their toddler. In addition, ECEC teachers and parents will be asked to fill in a 3-day food and physical activity record at baseline and 9 months follow-up (parents fill in the records for their toddler).

All measurements will be carried out by trained research staff and students of the Amsterdam University of Applied Sciences using Standard Operation Procedures. The study team is coordinated by 3 unblinded researchers (SM/MJ/NT) and 1 blinded researcher (MS).

Questionnaires, score forms and records will be coded to protect the privacy of the participants. Data from paper questionnaires and score forms will be handled into Microsoft Excel by double data-entry. Records will be entered in Microsoft Excel and checked by trained research staff and students of the Amsterdam University of Applied Sciences.

Table 1 provides a summary of the outcome measures for the study timepoints.

**Table 1:** Summary of the outcome measures for the study timepoints

TIMEPOINT	BASELINE			4 MONTHS					9 MONTHS		
	ECEC*	Toddlers	Parents		ECEC*	Toddlers	Parents		ECEC*	Toddlers	Parents
OUTCOME MEASURES											
*Demographic data*	X	X	X								
*Knowledge of Dutch food-based dietary guidelines*	X		X		X				X		X
*Attitude about healthy eating and physical activity*	X		X		X				X		X
*Food and activity practices*	X		X		X				X		X
*Level of confidence in promoting healthy eating and physical activity*	X				X				X		
*Anthropometry and body composition*	X	X			X				X	X	
*Dietary intake*	X	X							X	X	
*Physical activity level*	X	X							X	X	
*Role (prompts) on playground*					X				X		
*Amount and type of physical activity on playground*						X				X	
*Motor development*		X								X	
*Weight perception of weight status toddler*			X								X

*ECEC Early Childhood Education and Care teachers

#### Demographic data

The questionnaire for ECEC teachers and parents includes demographic questions to obtain general characteristics of the study population. Data of ECEC teachers, toddlers and parents will be collected, including the date of birth (age will be calculated in years (adults) and months (toddlers)), gender, country of birth (migration background) and level of education (proxy for socio-economic status, only applicable for ECEC teachers and parents).

#### Knowledge, attitude and practices regarding healthy eating and physical activity

The knowledge of ECEC teachers and parents will be assessed by 2 types of questions. Firstly, subjects will be asked to answer 10 statements on the Dutch food-based dietary guidelines with ‘True’, ‘False’ or ‘Don’t know’ [19]. Secondly, subjects will indicate in 3 separate questions if 5 food products (in total 15 food products) are ‘High’ or ‘Low’ in respectively added sugars, salt and fibres (or if they ‘Don’t know’) [20]. A sum score for Knowledge will be calculated; each correctly answered question yields one point with a maximum of 25 points.

Attitudes will be evaluated through individual statements regarding healthy eating and physical activity. The statements are compiled by the research staff. For example, ECEC teachers and parents will be asked to answer to what extent they do agree with the following statement: ‘I feel responsible for healthy nutrition- and physical activity patterns of my/the child(ren). A 5 point Likert-scale will be used. Answering options for the statements are 1) totally disagree (1 point), slightly disagree (2 points), neutral (3 points), slightly agree (4 points) or totally agree (5 points).

A modified version of the Child-care Food and Activity Practices Questionnaire (CFAPQ) [21] will be used to assess the practices of ECEC teachers related to food and physical activity. The original CFAPQ consists of 63 items (40 food-related and 23 activity-related items), divided over 12 CFAPQ scales (7 food-related and 5 activity-related scales). For the original CFAPQ, Gubbels et al. show a sufficient internal consistency in the CFAPQ scales with a Cronbach’s α ranging from 0.53 to 0.96 [21]. In PS@HW, a modified version of the CFAPQ will be used as not all CFAPQ scales are applicable for preschools. The PS@HW questionnaire for ECEC teachers includes the following 7 CFAPQ scales: Food-related Modelling/Encourage balance and variety, Food-related Teaching about nutrition, Food-related pressure to eat, Activity-related modelling, Activity-related psychological control, Activity-related teaching/autonomy support, Activity-related going outdoors and 1 single item about the availability of outdoor toys. Answering options for the questions are 1) totally disagree (1 point), slightly disagree (2 points), neutral (3 points), slightly agree (4 points), totally agree (5 points) or 2) never (1 point), rarely (2 points), sometimes (3 points), mostly (4 point), always (5 points). A mean score per CFAPQ scale will be calculated.

For parents, the Comprehensive Feeding Practices Questionnaire (CFPQ) [22] and the Preschooler Physical Activity Parenting Practices (PPAPP) instrument [23] will be used to assess parenting practices. The parenting practices questionnaires are both starting points for the CFAPQ (selected items of the questionnaires are converted to the child care setting by Gubbels et al.). The original CFPQ includes 12 CFPQ scales on food related practices. One CFPQ scale about restriction for weight control is considered to be too extensive for the purposes of PS@HW. Consequently, the following 11 CFPQ scales are incorporated in the PS@HW questionnaire for parents: Child control, Emotion regulation, Encourage balance and variety, Environment, Food as reward, Involvement, Modeling, Monitoring, Pressure, Restriction for health and Teaching about nutrition. The PPAPP concerns questions about encouraging and discouraging practices related to physical activity of children. The encouraging practices consist of 1 PPAPP scale (Engagement) and 2 single items (Not register the child for sports or dance due to lack of money and Having outdoor toys available for the child). The discouraging practices consist of 4 PPAPP scales, namely Promote inactivity, Promote screen time, Psychological control and Restriction for safety. The complete PPAPP is incorporated in the PS@HW questionnaire for parents. Answering options for the questions are 1) totally disagree (1 point), slightly disagree (2 points), neutral (3 points), slightly agree (4 points), totally agree (5 points) or 2) never (1 point), rarely (2 points), sometimes (3 points), mostly (4 point), always (5 points). A mean score per CFPQ and PPAPP scale will be calculated.

#### Level of confidence

The level of confidence of ECEC teachers related to promoting healthy eating and physical activity in toddlers, will be assessed using a confidence ruler [24]. ECEC teachers indicate, on a scale of 1 (not confident at all) to 10 (extremely confident), how confident they are in supporting toddlers (and their parents) in healthy eating and physical activity.

#### Anthropometry and body composition

In ECEC teachers and toddlers, body weight (kg) will be measured using a portable weighing scale (Seca 813) without shoes or heavy clothing. Moreover, body height (cm) will be measured with a portable stadiometer (Seca 213) without shoes. BMI (kg/m^2^) will be calculated. For toddlers, also the BMI z-score will be assessed using World Health Organization reference data (WHO Anthro). Weight status of the toddlers (underweight, normal weight, overweight and obesity) will be evaluated by reference data of Cole et al. [25]. In addition, the weight perception of parents concerning the weight status of their toddler will be examined via a question in the questionnaire for parents (answering options are: underweight, normal weight, overweight, severe overweight).

Total body resistance will be measured in ECEC teachers and toddlers by Bioelectrical Impedance Analysis (Bodystat 1500 MDD). The measured resistance will be used to calculate Total Body Water and subsequently Fat Free Mass and Fat Mass (kg/%) [26, 27]. For toddlers, hydration constants discussed by Fomon et al. will be used in the calculation of Fat Free Mass [28]. Furthermore, the Fat Mass Index (kg/m^2^) will be evaluated for toddlers.

#### Dietary intake

The dietary intake of ECEC teachers and toddlers will be estimated by 3-day food records. Food records are described to be useful in the estimation of dietary intakes in culturally diverse populations [29]. ECEC teachers will be asked to record their dietary intake per meal on 2 working days and 1 weekend day. Parents will be asked to fill in the 3-day food record for their toddler on 2 week days and 1 weekend day. Written and oral instructions will be given by the research staff. The intake of foods will be converted (by coding and converting household measures into grams) into energy and nutrient intake using the Dutch Food Composition Database 2016 [30] and a database with portion sizes [31].

#### Physical activity level

The physical activity level of ECEC teachers and toddlers will be determined using 3-day physical activity records. Self-report measures are commonly used to estimate physical activity levels [32] and are considered to be feasible for the purposes of PS@HW. ECEC teachers will be asked to record activities per 0.5 h on 2 working days and 1 weekend day. Parents will be asked to fill in activities for their toddler per 0.5 h during 2 week days and 1 weekend day. MET-scores will be assigned to each activity in order to calculate physical activity levels. For ECEC teachers, the 2011 Compendium of Physical Activities will be used as a reference for MET-scores [33]. For toddlers, a Youth Compendium of Physical Activities described by Butte et al. will be used [34]. In addition, ECEC teachers will be asked to wear an accelerometer (ActiGraph ActiTrainer) for 6 days to obtain objective data on their physical activity level. The data obtained by the ActiTrainers will be processed in the ActiLife Data Analysis Software version 5.10.0. Counts per minute will be assessed and the intensity of physical activity will be evaluated (sedentary to very vigorous).

#### The activating role of ECEC teachers and amount & type of physical activity in toddlers on the playground

To investigate the activating role of ECEC teachers regarding physical activity of toddlers on the playground, it was chosen to perform systematic observations on the playground of preschools. Furthermore, the amount and type of physical activity in toddlers on the playground will be systematically observed. As part of the ‘PLAYgrounds for Toddlers’ program, trained research assistants will perform the observations based on the SOPLAY protocol (physical activity levels during play time) [35]. This standardized protocol includes observations on the amount of physical activity, type of physical activity and aspects related to the physical environment (for example, weather conditions and provision of playground equipment). Before and after the ‘PLAYgrounds for Toddlers’ program, 2 observations on each playground will be performed.

#### Motor development

To assess motor development of toddlers, the Movement ABC2 test will be carried out (only applicable for toddlers of > 3.0 years) [36]. Standard Scores on each part of the Movement ABC2 assessment battery will be calculated. Thereafter, a Total Test Score for each toddler will be determined.

### Trial status

PS@HW is registered through the Netherlands Trial Register (NTR): NL5850.

### Planned statistical analysis

The sample size is based on a medium effect size (Cohen’s d: 0.50), a 1-sided alpha of 5% and a power of 80%, taken into account a design effect (mean cluster size = 3, ICC: 0.05) and a 10% drop-out rate.

Descriptive statistics (percentages, mean ± SD, or median (IQR)) will be used to describe the characteristic of the study population.

Mixed Model analyses will be used for the effect evaluation with primary and secondary outcome measures at the level of ECEC teachers, toddlers and their parents. Time, treatment (intervention or control group) and their interaction are fixed factors. Subject and location will be included as random factors to take into account the clustered data structure.

For the purpose of implementing the preschool-based intervention on organizational level, a process evaluation focusing on the acceptance, satisfaction and improvements of the intervention will be performed.

## Discussion

This study aims to evaluate the effect of a preschool-based intervention for ECEC teachers. It is hypothesized that the intervention will increase the knowledge, attitude and practices of ECEC teachers regarding healthy eating and physical activity and consequently the level of confidence in promoting healthy eating and physical activity of toddlers. The preschool-based intervention addresses the call for early intervention to prevent overweight and obesity and to minimize health inequalities.

Strengths include the randomized controlled study design and multi-component and multi-level character of the intervention. Moreover, the diverse study population is a strength. However, the study is limited in the ability to generalize the findings, as only preschools of 1 child care organization in Amsterdam are included.

## Abbreviations

BMI: Body Mass Index
CFAPQ: Child-care Food and Activity Practices Questionnaire
CFPQ: Comprehensive Feeding Practices Questionnaire
ECEC: Early Childhood Education and Care
PPAPP: Preschooler Physical Activity Parenting Practices
PS@HW: PreSchool@HealthyWeight
